# Diagnostic value of routine dental radiographs for predicting the mandibular canal localization validated by cone‐beam computed tomogram measurements

**DOI:** 10.1002/cre2.639

**Published:** 2022-08-08

**Authors:** Bernhard Wiechens, Phillipp Brockmeyer, Tayhan Sevinc, Georg Hoene, Henning Schliephake, Wolfram Hahn

**Affiliations:** ^1^ Department of Orthodontics University Medical Center Göttingen Göttingen Germany; ^2^ Department of Oral and Maxillofacial Surgery University Medical Center Göttingen Göttingen Germany; ^3^ Private practice Bonn Germany; ^4^ Private practice Göttingen Germany

**Keywords:** cephalometry, cone‐beam computed tomography, mandibular canal, radiography, dental, radiography, panoramic

## Abstract

**Objectives:**

To test the hypothesis that routine dental radiographs can be used to draw conclusions regarding the mandibular canal (MC) localization.

**Material and Methods:**

A total of 108 radiographs (36 orthopantomograms [OPTs], 36 lateral cephalograms [LCs], and 36 cone‐beam computed tomograms [CBCTs]) of 36 patients were analyzed. Statistical analysis included all cephalometric parameters obtained by OPTs, LCs, and CBCTs. Potential influencing parameters were calculated using linear and logistic regression with a backward removal algorithm. For predictability of MC localization, parameters were correlated using Pearson's correlation.

**Results:**

The MC ran along the lingual half (*n* = 24) twice as often as in the buccal half (*n* = 12) in the population studied. The position was always symmetrical contralaterally. No sex‐specific influence was observed (*p* = .34). Lingual and buccal MC courses were statistically significantly correlated to increased and decreased jaw angles (LC/OPT), respectively (*p* = .003; *r* = −.48/*p* = .010; *r* = −.42). An increased jaw angle was significantly correlated with a more cranial MC position (*p* = .013; *r* = −.41); a deep and distal bite position was significantly correlated with a caudal and buccal MC position (*p* = .004; *r* = −.47/*p* = .001; *r* = .57). Moreover, an increase of 1 point in the Hasund score predicted an increased probability of a buccal MC position by 18.6%. The jaw angle analyzed in OPT and LC images were positively correlated (*r* = .89, *p* < .001).

**Conclusions:**

Routine dental radiographs provide informative guidance on the location of the MC in the vertical and transverse levels. This finding could be used in the initial consultation and treatment planning to consider more invasive diagnostic methods further down the line.

## 1 INTRODUCTION

Lateral cephalograms (LCs) and orthopantomograms (OPTs) are indispensable for orthodontic diagnosis and treatment planning (American Academy of Oral and Maxillofacial Radiology, 2013; Rischen et al., 2013). Cephalometric analysis and the relationships among the jaws, dentition, and soft tissues provide essential information about the pathogenesis of dental malocclusions (American Academy of Oral and Maxillofacial Radiology, 2013; Rischen et al., 2013). However, heretofore, there is no consensus on the diagnostic value or justifiable indication of LCs and OPTs, as some studies critically question the validity of cephalometric analysis and the influence on orthodontic treatment decisions. This is primarily because of the fact that no clear and universal definition of basic radiological diagnostics for orthodontic treatment can be found in the extant literature (American Academy of Oral and Maxillofacial Radiology, 2013; Devereux et al., 2011; Durão et al., 2015; Nijkamp et al., 2008; Rischen et al., 2013). Radiological diagnostics are associated with radiation exposure of the patient, which can be considerable even at low doses (Brenner, 2009; Ghorbani & Fardid, 2021). Particularly in the case of children, who make up the largest proportion of patients undergoing orthodontic treatment, it must therefore be well considered whether and which radiological technique should be used (Devereux et al., 2011). The aforementioned routine dental radiological imaging techniques (LCs and OPTs) provide a wealth of information in terms of incidental findings that are rarely recorded, interpreted, and used for further treatment in everyday practice (Bondemark et al., 2006). Although not uniformly defined internationally, LC and OPT imaging in orthodontic initial diagnostics for basic treatment represent the break‐even point between diagnostic information content and minimum necessary radiation exposure according to the ALARA principle (as‐low‐as‐reasonably achievable principle) (Kapetanović et al., 2021). Nevertheless, due to the continuous technical progress of imaging techniques, the further development of supporting software and the considerably higher information content of three‐dimensional imaging, it is currently necessary to continuously examine whether the indication should not be made in favor of cone‐beam computed tomogram (CBCT) imaging (Kapetanović et al., 2021). Particularly with regard to questions concerning the inferior alveolar nerve for planning wisdom tooth extractions or the insertion of temporary anchorage devices, two‐dimensional imaging seems to be at its limit, although conclusions seem to be possible (Matzen & Wenzel, 2015; Matzen et al., 2020; Patil et al., 2013). Haas et al. (2016) remarked in a systematic review and meta‐analysis that the diagnosability of the nerve position in OPT should be investigated, as some studies have shown a prevalence of 1% in OPTs (Correr et al., 2013; de Oliveira‐Santos et al., 2012), whereas in CBCT scans, nerve position abnormalities were found in up to 30% cases (Haas et al., 2016). It can be stated that the information density of CBCTs is superior to two‐dimensional imaging and provides more detailed information about the course of the mandibular canal (MC) as well as the inferior alveolar nerve, especially with regard to the presented examination focus (Kapetanović et al., 2021; Lo Giudice et al., 2021). Recent studies by Lo Giudice et al. (2021) and Leonardi et al. (2021) impressively demonstrated the potential resolution and detectability of even the smallest hard tissue changes by CBCT imaging and again established its superiority over two‐dimensional imaging (Kapetanović et al., 2021). Therefore, the present study was not intended to question the superiority of three‐dimensional imaging, but rather to take advantage of the high information content to possibly identify additional sources of information in two‐dimensional imaging that seem to be unused to date. Therefore, the diagnostic value of routine two‐dimensional dental radiographs (OPTs and LCs) should be assessed in relation to MC position. Furthermore, it should be evaluated whether the results can be used in initial consultation and treatment planning to expand the information content of two‐dimensional imaging based on the findings of CBCT imaging.

## 2 MATERIALS AND METHODS

### 2.1 Patients

This retrospective analysis was conducted in accordance with the principles of the Declaration of Helsinki. The Ethics Committee of the University Medical Center Göttingen approved the study protocol (application number DOK_342_2015). Written consent to use radiographs for scientific purposes was obtained from each patient. The sample size of 34 subjects was determined with G*Power (v.3.1.9.2; University of Düsseldorf, Düsseldorf, Germany) by applying a significance level of .05, a power of 0.9, and a large effect size of 0.5. The effect size was calculated for clinically relevant correlations between imaging modalities of at least *r* = .5 (Navarro et al., 2013). A total of 108 radiographs (36 OPTs, 36 LCs, and 36 CBCTs) of 36 patients, which were indicated within the scope of dental, orthodontic, and oral and maxillofacial surgery treatments were retrospectively analyzed. In addition to the presence of all three imaging modalities, study inclusion required skeletal maturity and circumferent‐supported natural dentition. The presence of craniofacial syndromes, already performed maxillomandibular advancement, bone trauma, or diseases of the jaw bases, led to study exclusion. CBCTs were obtained during maxillofacial surgical diagnostics and therapy planning using a PaX Zenith 3D (Orange Dental, Biberach an der Riß, Germany; field of view of 240 × 190 mm, 24 s, 0.3 voxel, 120 KVP, and 6 mAs). OPTs (14.1 s, 62–66 KVP, and 14–16 mA), and LCs (9 s, 77–80 KVP, and 14–15 mA), with a 10% rate of magnification and with patients placed at 1.5 m away from the unit were obtained for orthodontic cephalometric analysis and treatment planning using an Orthophos XG Plus (Sirona Dental Systems GmbH, Bensheim, Germany). The patient population consisted of 20 women and 16 men, with ages ranging from 18 to 51 years (mean age: 25.8 years). The average skeletal parameters of the study population are encapsulated in Table 1. All data were collected in the period from March 2019 to July 2020 and analyzed by two independent experienced investigators. Study inclusion criteria were completed skeletal maturity and a circumferent‐supported natural dentition. Exclusion criteria were craniofacial syndromes, already performed maxillomandibular advancement, bone trauma, and diseases of the jaw bases.

**Table 1:** Descriptive statistics for all measurements of LC, OPT, and CBCT

LC						
Parameter	Unit	*n*	Mean	Minimum	Maximum	SD
SNA	°	36	80.16	68.70	90.90	5.26
SNB	°	36	78.16	62.70	94.25	8.28
ANB	°	36	1.88	−14	12.60	6.23
ML‐NSL	°	36	33.26	10.05	51.25	10.43
NL‐NSL	°	36	9.11	1.20	21.55	4.55
ML‐NL	°	36	24.15	2.15	42.65	9.22
NSBa	°	36	131.89	117.85	168	9.30
Gn‐Go‐Ar	°	36	125.33	103.05	138.95	8.57
Index	%	36	77.36	64	101	7.97
Hasund	score	36	2.47	−12	17	6.96
OPT						
Mand.Ang.	°	36	127.50	111.08	140.40	7.20
RT7	mm	36	13.19	7.50	20.53	3.28
CBCT						
MN1	mm	36	11.09	5.55	16.60	2.63
MN2	mm	36	10.26	4.95	16.50	3.12
MN3	mm	36	12.30	7.05	22.00	3.90

*Note*: Values of SNA, SNB, ANB, ML‐NSL, NL‐NSL, ML‐NL, NSBa, Gn‐Go‐Ar and Mand.Ang. are given in degrees. Index is given in percent. Hasund's value is given in ordinal score; RT7 and MN1–3 are given in millimeters.

Abbreviations: ANB, A‐point‐nasion‐B‐Point angle; CBCT, cone‐beam computed tomogram; Gn‐Go‐Ar, Gnathion‐Gonion‐Articulare angle; LC, lateral cephalograms; Mand.Ang., mandibular angle; ML‐NL, Mandibular‐Line‐Nasal‐Line angle; ML‐NSL, Mandibular‐Line‐Nasion‐Sella‐Line angle; NSBa, nasion‐sella‐basion angle; OPT, orthopantomograms; SNA, sella‐nasion‐A‐point angle; SNB, sella‐nasion‐B‐point angle.

### 2.2 Radiological evaluation

**Figure 1:**
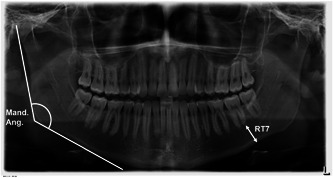
Orthopantomogram with acquired measurements. Distance of root tip of second molar (RT7) to mandibular base and jaw angle (Mand.Ang.).

All data were analyzed by two independent examiners with experience in the field of cephalometric analysis. The subsequent test for normal distribution was performed with Shapiro–Wilk tests. Subsequently, interrater reliability was confirmed using Bland–Altman plots (Table 2). In each OPT, the mean value of both jaw angles (Mand.Ang.) was determined. In addition, the average distances of the root tips of the second molars of both quadrants (RT7) to the mandibular base were measured (Figure 1). All OPTs were evaluated using the SIDEXIS XG® software (Sirona Dental Systems GmbH, Bensheim, Germany).

**Table 2:** Results of Bland–Altman plots on PR, LCR, and CBCT evaluation of both independent investigators

Parameter	Unit	Lower limit of agreement	Upper limit of agreement
Mand.Ang.	°	−1.85	1.89
SNA	°	−1.14	1.04
SNB	°	−1.51	2.41
ANB	°	−3.25	2.49
ML‐NSL	°	−1.55	1.78
NL‐NSL	°	−0.92	1.19
ML‐NL	°	−1.44	1.4
NSBa	°	−3.91	3.44
Index	%	−2.31	1.84
Gn‐Go‐Ar	°	−2.06	2.04
Hasund	score	−1.39	1.34

*Note*: Angles are given in degrees (°), index in percent (%), and Hasund as score.

Abbreviations: ANB, A‐point‐nasion‐B‐point angle; CBCT, cone‐beam computed tomogram; Gn‐Go‐Ar, gnathion‐gonion‐articulare angle; LC, lateral cephalograms; Mand.Ang., mandibular angle; ML‐NL, mandibular‐line‐nasal‐line angle; ML‐NSL, mandibular‐line‐nasion‐sella‐Line angle; NSBa, Nasion‐Sella‐Basion angle;PR, panoramic radiographs; SNA, Sella‐Nasion‐A‐Point angle; SNB, Sella‐Nasion‐B‐Point angle.

**Figure 2:**
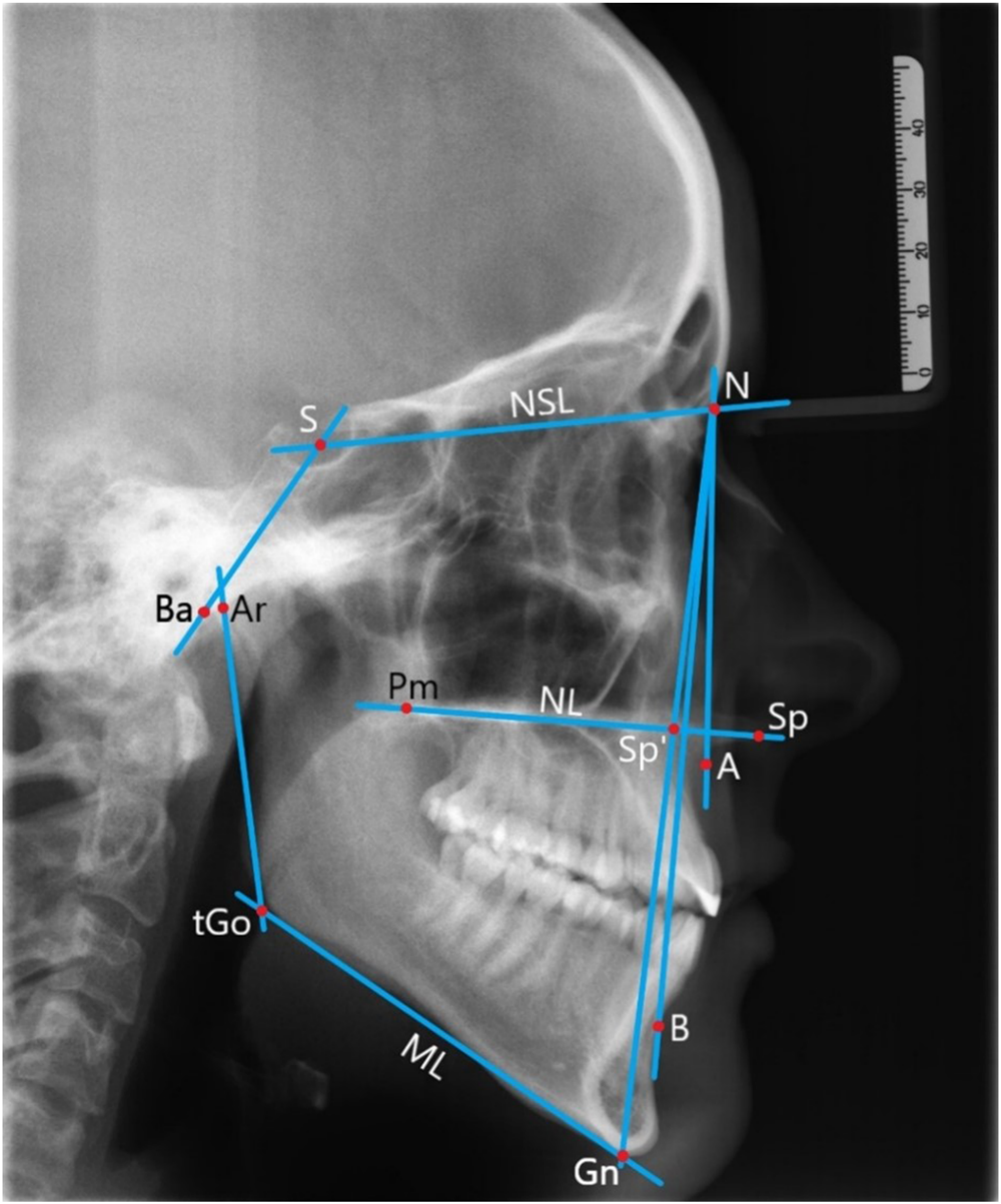
Lateral cephalogram with cephalometric variables plotted: SNA angle, SNB angle, ANB angle, ML‐NSL angle, NL‐NSL angle, ML‐NL angle, NSBa angle, Gn‐Go‐Ar angle, and Index = $\frac{N‐Sp\;\prime }{Sp\;\prime ‐Gn}\times 100$ ANB, A‐point‐nasion‐B‐point angle; Gn‐Go‐Ar, gnathion‐gonion‐articulare angle; ML‐NL, mandibular‐line‐nasal‐line angle; ML‐NSL, mandibular‐line‐nasion‐sella‐line angle; NSBa, nasion‐sella‐basion angle; SNA, sella‐nasion‐A‐point angle; SNB, sella‐nasion‐B‐point angle.

In each LC, eight different angles were measured (Figure 2). The ratio of the anterior facial heights (index) was determined using Hasund's cephalometric analysis, and a structural analysis of the mandible was performed according to the method proposed by Björk and modified by Segner and Hasund (Björk, 1969; Lenza et al., 2015; Segner & Hasund, 2003). The entire cephalometric analysis was performed using the ivoris® analyze software (Computer Konkret AG, Falkenstein, Germany).

**Figure 3:**
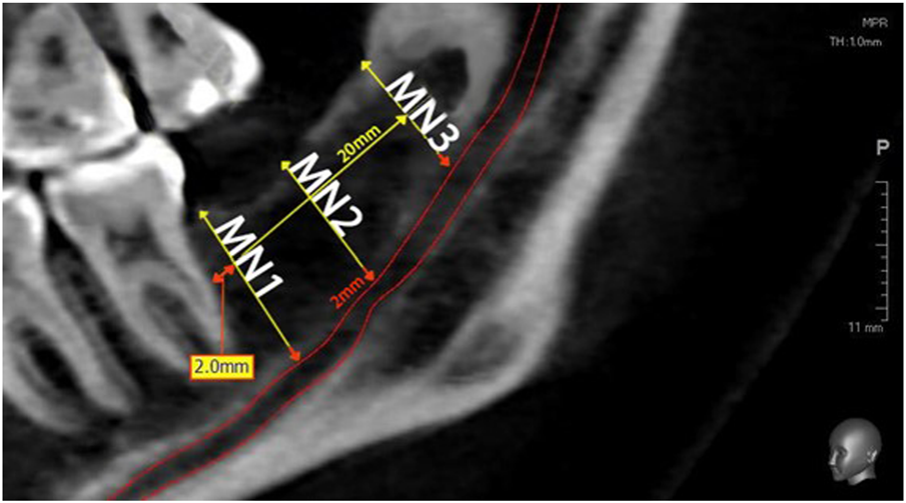
Sagittal section of retromolar region in cone‐beam computed tomogram image with mandibular canal marked in red and measuring points marked in yellow

**Figure 4:**
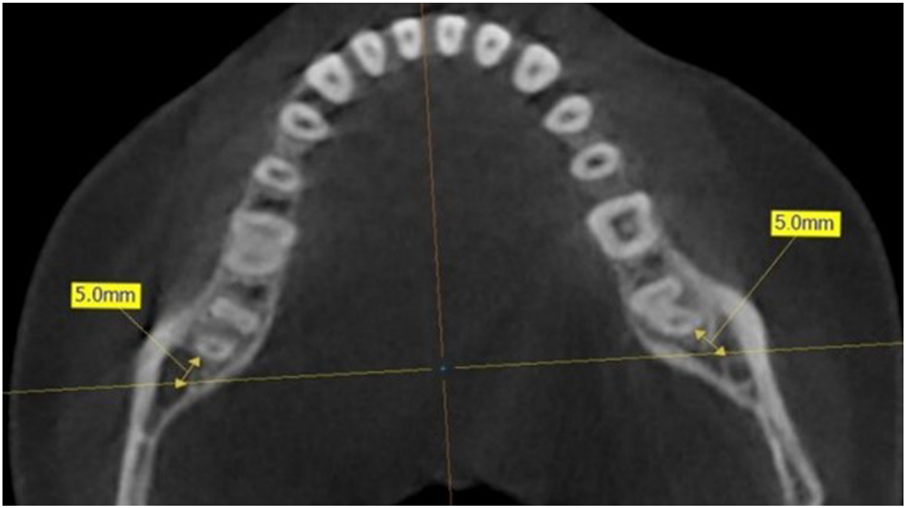
Axial view of the mandible in cone‐beam computed tomogram image with a marked distance of 5 mm distal to the last molar. Coronal view was adjusted using corresponding distance markings.

**Figure 5:**
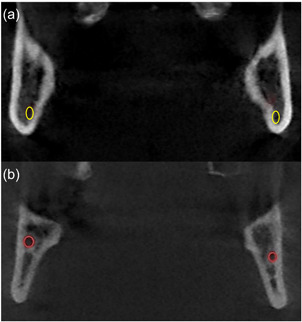
Coronal view of the mandible in cone‐beam computed tomogram image. Lingual (a) and buccal (b) mandibular canal is marked in red.

All CBCT scans were analyzed using the Ez3D Plus® software (Vatech Company, Hwaseong, Korea). The vertical distance between the MC and the occlusal bone surface was determined at three different measuring points (MN1–3) over a length of 20 mm, starting distal in direction to the second mandibular molar. The measuring points MN1–3 were thus located at a distance of exactly 10 or 20 mm from each other (Figure 3). In addition to the vertical position of the MC, the transverse position was examined and divided into two groups. For this purpose, a distance of 5 mm distal to the second mandibular molar was marked in the axial view of the CBCT scans (Figure 4). According to this distance, a coronal view could be set, which was used to assess the nerve position with subsequent software‐assisted drawing (Ez3D‐i Draw Canal; Vatech Company, Hwaseong, Korea) of the mandibular canal (Figure 5). Data were divided into two groups depending on whether the MC ran in the lingual or buccal half of the mandibular bone (Figure 5 and Table 3).

**Table 3:** *T* tests for examining cephalometric measurements as the independent variable and mandibular canal course as the dependent variable

Parameter	Unit	Buccal nerve course (mean)	Lingual nerve course (mean)	Correlation coefficient	*p* Value	Buccal course (*n*)	Lingual course (*n*)
NSBa	°	136.36	129.65	0.35	.039*	12	24
Gn‐Go‐Ar	°	119.65	128.18	−0.48	.003**	12	24
Mand.Ang.	°	123.25	129.62	−0.42	.010*	12	24
Hasund	score	6.83	0.29	0.45	.006**	12	24

*Note*: Significance level was *p* < .05. Only statistically significant parameters are given.

Abbreviation: Mand.Ang., mandibular angle.

* *p* < .05;

** *p* < .01.

### 2.3 Statistical analysis

Statistical analysis was performed using Student's *t* test, *χ* ^2^ test, linear regression, logistic regression with backward removal algorithm, and Pearson's correlation. All tests were performed at a significance level of *α* = 5%, using the statistical software STATISTICA® (StatSoft Europe GmbH, Hamburg, Germany) and SPSS® (IBM Corporation, New York, USA).

## 3 RESULTS

### 3.1 Interrater variations

Table 2 summarizes the results of the Bland–Altman plots. The largest range of interrater variation was found for the NSBa parameter, where 95% of the measurement differences between the two raters were in the range of −3.91 and 3.44 mm. Considering the other parameters, a low interrater variability could be assumed.

### 3.2 Descriptive statistics

The averaged skeletal configuration of the patients showed a bimaxillary orthognathic configuration, with sagittal and vertical neutral jaw relation, neutral maxillary and mandibular base inclination, neutral skull base inclination, and neutral index and jaw angle (Table 1). The mean inferior bone height was 25.39 mm, and the mean vertical MC position ranged from 10.26 mm (MN1) to 12.30 mm (MN3).

### 3.3 Significance of cephalometric parameters for MC localization

Table 3 presents the results of the applied *t* test to analyze the correlations between the cephalometric measurements of the LC/OPT images and the buccal or lingual MC position according to the CBCT scans. The cephalometric angle NSBa (LC) and mandibular angles (Gn‐Go‐Ar/LC and Mand.Ang./OPT) correlated significantly with a corresponding MC localization (*p* = .039; *r* = .35/*p* = .003; *r* = −.48/*p* = .010; *r* = −.42, respectively). Thus, a decreased NSBa angle and increased mandibular angles (Gn‐Go‐Ar/LC and Mand.Ang./OPT) predicted a lingual MC position, while an increased NSBa angle and decreased mandibular angles indicated a buccal MC position. The extent of Hasund's score measured in the LC confirmed this relationship with a strongly significant expression and medium effect size correlation (*p* = .006; *r* = .45).

### 3.4 Relevance of jaw angle and bite relation for nerve localization

**Figure 6:**
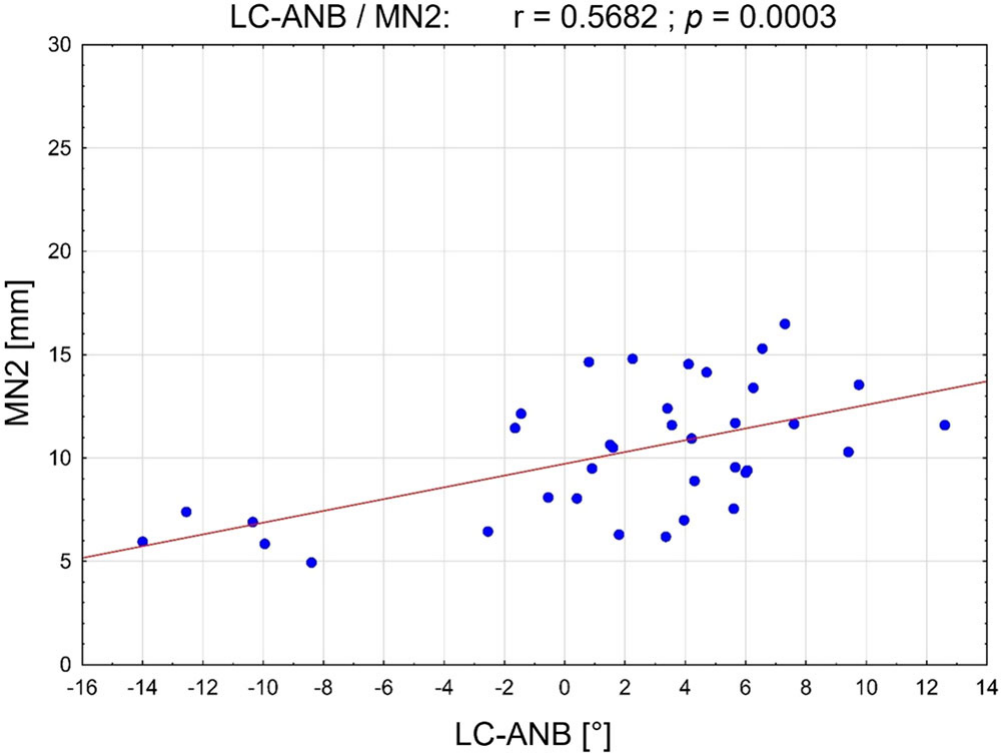
Scatterplot of Pearson's correlation between ANB (°) (measured on lateral cephalograms) and vertical position of mandibular canal MN2 (mm) (measured on cone‐beam computed tomogram). ANB, A‐point‐nasion‐B‐point angle; MN, vertical distance between mandibular canal measuring‐point and occlusal cortical border.

Table 4 summarizes the significant results of Pearson's correlation analysis between the LC and CBCT measurements. All measurement points of the vertical nerve position in the CBCT scans were significantly negatively correlated with the sagittal mandibular position according to the LC (SNB). In retral mandibular positions (decreased SNB), increased distances between the nerve position and the occlusal cortical bone could be determined. Accordingly, the nerve position was caudal in retral located mandibles and cranial in anteriorly located mandibles. The negative correlation was significant for the measurement points MN1 and MN3 (*p* = .040/*r* = −.34 and *p* = .012/*r* = −.41, respectively) and strongly significant for measurement point MN2 (*p* = .006/*r* = −.45). The correlation analysis of the sagittal skeletal configuration confirmed the previously described relationship according to which increased deviations of the mandibular bases in the sagittal plane (ANB) were associated with an increase of the measurement points MN1–3 (Figure 6). The positive correlation was strongly significant for measurement points MN1 and MN2 (*p* = .003/*r* = .49; *p* = .001/*r* = .57) and significant for point MN3 (*p* = .044/*r* = .34). Measuring point MN3 was negatively correlated with the jaw angle (*p* = .004/*r* = −.47) and positively correlated with the Hasund score (*p* = .002/*r* = .50). A decreased (increased) jaw angle and an increased (decreased) Hasund score thus allowed caudal nerve position to be detected and vice versa.

**Table 4:** Pearson correlations between LC and CBCT measurements of retromolar region, as well as LC and OPT measurements

Correlations between LC and CBCT measurements				
LC	Unit	CBCT (retromolar)	Correlation coefficient	*p* Value
SNB	°	MN1	−0.34	.040*
		MN2	−0.45	.006**
		MN3	−0.41	.012*
ANB	°	MN1	0.49	.003**
		MN2	0.57	.001**
		MN3	0.34	.044*
Gn‐Go‐Ar	°	MN3	−0.47	.004**
Hasund	score	MN3	0.50	.002**

Correlations between LC and OPT measurements				
LC	Unit	OPT	Correlation coefficient	*p* Value
Gn‐Go‐Ar	°	Mand.Ang.	0.89	<.001***

*Note*: Hasund score ranging from −18 to +18.

Abbreviations: ANB, A‐point‐nasion‐B‐point angle; ‐articulare angle; CBCT, cone‐beam computed tomogram; LC, lateral cephalograms; Mand.Ang., mandibular angle; OPT, orthopantomograms; SNB, sella‐nasion‐B‐point angle.

* *p* < .05;

** *p* < .01;

*** *p* < .001.

### 3.5 Relationship between the mandibular base and nerve localization

**Figure 7:**
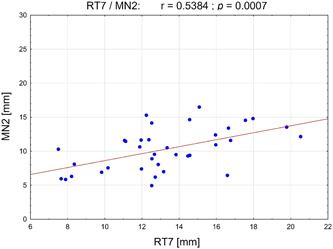
Scatterplot of Pearson's correlation between distance RT7 (mm) (measured on orthopantomograms) and vertical position of mandibular canal MN2 (mm) (measured on cone‐beam computed tomogram)

The RT7 value (OPT) was correlated positively with a large effect size for vertical MC positions on the CBCT scans (MN1, MN2, and MN3: *p* = .002/*r* = .51; *p* = .001/*r* = .54, and *p* = .013/*r* = .41, respectively) (Table 5 and Figure 7). Thus, an increased RT7 distance suggested an increase in MN1–3 measurements on CBCT, suggesting a more caudal nerve localization. This observation was supported by the negative correlation of the Mand.Ang. and the most posterior measurement point MN3 (CBCT). With reduced jaw angles, distances significantly increased, and thus more caudal nerve positions were found (*p* = .013/*r* = −.41).

**Table 5:** Pearson's correlations between OPT measurements and CBCT measurements in retromolar region

OPT	Unit	CBCT (retromolar)	Correlation coefficient	*p* Value
RT7	mm	MN1	0.51	.002**
		MN2	0.54	.001**
		MN3	0.41	.013*
Mand.Ang.	°	MN3	−0.41	.013*

Abbreviations: CBCT, cone‐beam computed tomogram; Mand.Ang., mandibular angle; OPT, orthopantomograms.

* *p* < .05;

** *p* < .01.

### 3.6 Relevance of mandibular structure for MC localization

The influence of all parameters on the observed correlations of the MC position was tested using logistic regression analysis with a backward removal algorithm. For a buccal nerve position, the analysis revealed an odds ratio of 1.186 for Hasund's score. Thus, the relative chance of the MC being positioned in the buccal half of the mandible at the position examined increased by 18.6% when the Hasund score increased by 1 (*p* = .020).

### 3.7 Correlation between LC and OPT measurements

**Figure 8:**
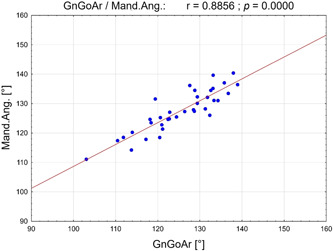
Scatterplot of Pearson's correlation between Gn‐Go‐Ar (°) (measured on lateral cephalograms) and mandibular angle (°) (measured on orthopantomograms). Gn‐Go‐Ar

A strongly positive correlation between the jaw angles measured in LC and OPT could be observed (*r* = .89; *p* < .001; Table 4 and Figure 8).

## 4 DISCUSSION

This study investigated the correlations of cephalometric analyses on routine two‐dimensional radiographs (OPT and LC) with data obtained from CBCT scans to predict the mandibular canal position. The results indicated a close correlation of various parameters of the less radiation‐intensive standard techniques with those of CBCT scans. Considering Cohen's interpretation of effect sizes (Cohen, 1992, 2013), a medium to strong effect was found for all parameters investigated. The smallest but still medium effect size was found for the LC parameter NSBa, which may be explained by the fact that this angle refers to the inclination of the cranial base and is thus to be regarded as a rather peripheral marker. Nevertheless, the significant correlation of the skull base inclination on the development of Class II and III malocclusions has been investigated well (Bhattacharya et al., 2014; Chin et al., 2014); therefore, the correlations detected in the present study with regard to the bucco‐lingual MC position can be considered as an interesting addition. Furthermore, it was observed that the effect strengths in the present study always increased when parameters in direct topography to the MC were examined. Thus, effect sizes of (*r* = −.48 and −.42) were found for the jaw angles Gn‐Go‐Ar (LC) and Mand.Ang. (OPT), respectively, which according to Cohen were already in the threshold from medium to strong effect sizes. The parameters ANB, Gn‐Go‐Ar, Hasund, and RT7 for the vertical MC position yielded the strongest expressions for the MC localization, whereby a large effect size was found for RT7, Hasund, and ANB. Combining the results of the present study with previously published findings, an extended benefit of OPT and LC imaging can be formulated due to the additional informative value with regard to MC localization (Bhattacharya et al., 2014; Bruks et al., 1999; Chin et al., 2014; Kapetanović et al., 2021; Mattick et al., 1999). This could be of particular interest during the initial consultation and treatment planning and to justify more radiation‐intensive three‐dimensional imaging techniques. Using the dedicated knowledge about the individual cephalometry of the patient (LC) and the configuration of the mandibular base (OPT), it was observed that relevant statements about the MC position can already be made in the initial diagnostic process. Compared to the more radiation‐intensive three‐dimensional methods, the combination of both two‐dimensional imaging techniques yielded information with a high correlation. In addition, further correlations could be determined by the structural analysis method, according to Björk (Björk, 1969; Lenza et al., 2015). For the first time, direct relationships between the morphological characteristics of the mandible and MC position could be determined in a specific question (Bremen & von, Pancherz, 2005). The diagnostic benefit lay in the combination of findings, which can already find special application in the initial orthodontic therapy planning. Skeletal anchorage devices are being used more and more frequently (Anhoury, 2013; Poletti et al., 2013; Sugawara et al., 2008; Yanagita et al., 2011). The retromolar region not only offers biomechanically favorable conditions for extensive en masse dental arch retractions (Anhoury, 2013; Poletti et al., 2013) but also has the closest positional relationship to the MC (Anhoury, 2013; Poletti et al., 2013; Sugawara et al., 2008; Yanagita et al., 2011), representing a high risk for nerve injuries (Denio et al., 1992). As described earlier (Denio et al., 1992), nerve injuries could be avoided without the use of radiation‐intensive three‐dimensional imaging.

Moreover, the osteotomy of impacted third molars can lead to an inferior alveolar nerve (IAN) damage (Guerrero et al., 2014). Guerrero et al. (2014) investigated the incidence of IAN lesions during third molar extraction using OPT or CBCT navigation and did not find significant differences in postoperative complications between both techniques. At the same time, however, the authors pointed out that the bucco‐lingual nerve position can be easily determined using CBCT scans, which is a clear advantage of three‐dimensional imaging. The results of the present investigation revealed that a statement about the bucco‐lingual MC position can also be made by combining the findings of the LC. Other studies have addressed the significance of OPT compared with CBCT but never combined the investigation with LCs. A systematic review and meta‐analysis by Reia et al. (2021) found accuracy values for IAN position using CBCT of 95.1% for sensitivity (*p* = .666) and 64.4% for specificity (*p* < .001) and for OPT 73.9% (*p* = .101) to 24.8% (*p* < .001). The authors remarked that both techniques reliably detected the IAN position, but the CBCT examination achieved better performance in predicting the IAN position during surgery.

Regarding the accuracy of texture‐analytical approximations using CTs or CBCTs to predict soft tissue structures, like the mandibular nerve as performed in the present study, there is currently disagreement, which mainly relates to the analysis of gray‐scale textures due to differences in image acquisition protocols, changes in quantitation, and reconstruction algorithms (Caramella et al., 2018). In contrast, Bahrampour et al. (2016) reported that automated software programs for MC localization had clear advantages over conventional methods in terms of rapidity and accuracy. However, Azcárate‐Velázquez et al. (2015) summarized in their study of 11 cadaver mandibles that although CBTC was the best diagnostic tool currently available, it was still unreliable compared to actual results, as they found average discrepancies of 1.15 mm in the thickness of the vestibular bone wall covering the MC and an average of 0.3 mm in the thickness of the inferior dental nerve. Despite the fact that CBCT was certainly referenced as the most reliable and accurate imaging in the present work, a degree of standard error can also be assumed here on the basis of current studies and the scientific consensus, which, however, was not to be investigated in the present study. The results of the presented investigation on MC position, therefore, nevertheless suggest a correction, especially with regard to the sensitivity of OPTs due to their ability to predict nerve position in the bucco‐lingual direction. In contrast, Ghaeminia et al. (2009) concluded that CBCTs were not more accurate in predicting MC position during third molar removal, but clarified the possibility of three‐dimensional imaging of the third molar root to the MC. However, the authors also affirmed the possibility of using coronal slices to perform a bucco‐lingual assessment of the mandibular channel on CBCT scans to identify cases in which a lingually placed MC is at risk during surgery.

## 5 CONCLUSION

Strong correlations were found between cephalometric parameters in OPT and LC images and data from CBCT scans regarding the position of the mandibular canal. Considering these results and taking into account the limitations of all imaging modalities, it can be safely said that the diagnostic value of two‐dimensional imaging needs to be re‐evaluated and could serve as a decision support for the future indication of more comprehensive three‐dimensional imaging modalities, although the information value of CBCTs will always be superior for the purposes presented.

## AUTHOR CONTRIBUTIONS

*Conceptualization*: Wolfram Hahn and Tayhan Sevinc. *Methodology*: Wolfram Hahn and Tayhan Sevinc. *Formal analysis and investigation*: Wolfram Hahn, Tayhan Sevinc, Bernhard Wiechens, and Phillipp Brockmeyer. *Writing—original draft preparation*: Bernhard Wiechens and Phillipp Brockmeyer. *Writing—review and editing*: Henning Schliephake and Georg Hoene.

## CONFLICT OF INTEREST

The authors declare no conflict of interest.

## ETHICS STATEMENT

The Ethics Committee of the University Medical Center Göttingen, Germany approved this study (application number DOK_342_2015). All patients gave their written informed consent to participate.

## Data Availability

The data underlying this article are available from the corresponding author on reasonable request.
